# Iterative Approximation of Basic Belief Assignment Based on Distance of Evidence

**DOI:** 10.1371/journal.pone.0147799

**Published:** 2016-02-01

**Authors:** Yi Yang, Yuanli Liu

**Affiliations:** SKLSVMS, School of Aerospace, Xi’an Jiaotong University, Xi’an, Shaanxi, China 710049; Southwest University, CHINA

## Abstract

In the theory of belief functions, the approximation of a basic belief assignment (BBA) is for reducing the high computational cost especially when large number of focal elements are available. In traditional BBA approximation approaches, a focal element’s own characteristics such as the mass assignment and the cardinality, are usually used separately or jointly as criteria for the removal of focal elements. Besides the computational cost, the distance between the original BBA and the approximated one is also concerned, which represents the loss of information in BBA approximation. In this paper, an iterative approximation approach is proposed based on maximizing the closeness, i.e., minimizing the distance between the approximated BBA in current iteration and the BBA obtained in the previous iteration, where one focal element is removed in each iteration. The iteration stops when the desired number of focal elements is reached. The performance evaluation approaches for BBA approximations are also discussed and used to compare and evaluate traditional BBA approximations and the newly proposed one in this paper, which include traditional time-based way, closeness-based way and new proposed ones. Experimental results and related analyses are provided to show the rationality and efficiency of our proposed new BBA approximation.

## Introduction

The theory of belief functions [1], also called Dempster-Shafer theory (DST), has many advantages in uncertainty modeling and reasoning [2, 3]; however, it has also been argued due to its limitations [4, 5]. One of the limitation is the high computational cost encountered in the procedure such as the evidence combination, conditioning, marginalization, and belief and plausibility degrees evaluation [1, 6], especially when large amount of focal elements are available. This will confine the use of DST in practical applications.

The approaches of BBA approximation were then proposed to deal with the high computational cost encountered. The BBA approximation aims to obtain a simpler BBA by removing some focal elements. In existing works, such a removal of focal elements was implemented according to three different criteria related to the focal element’s own characteristics. The first criterion is the mass assignment of a focal element, where the focal elements with smaller mass assignments are deemed unimportant, which are first removed. *k* − *l* − *x*[7], Summarization [8] and *D*1 [9] are representatives for this criterion. The second criterion is the cardinality of a focal element, where the focal elements with larger cardinalities, which might cause more computational cost, are first removed. *k*-additive approximation [10] is a representative of the 2nd criterion. The third criterion is to jointly use the mass assignments and cardinality of focal elements to determine which focal element should be first removed. Denœux’s inner and outer approximations [11], our proposed rank-level fusion based approximations [12], and our proposed non-redundancy based BBA approximations [13] follow the 3rd criterion.

Although all the available work referred above are rational and make sense to some extent, they still have their limitations. For example, the 1st and 2nd criteria only emphasize one aspect of the characteristic of focal elements. The 3rd criterion is the right direction, which jointly uses the mass assignments and cardinalities. This will make the related approximations more comprehensive, which means that they consider multiple aspects and thus avoid to be one-sided. The available works based on the 3rd criteria are good attempts; however, the joint use is still an open problem, whose theoretical strictness and the generalization capability need further verification. To approximate the BBA more effectively, in this paper, we propose another approximation according to the 3rd criterion (i.e., the joint use of the 1st and 2nd) from a different angle. We directly start from the performance evaluation criterion of BBA approximations to design new approaches. Besides the computational cost, the distance between the approximated BBA and the original one, which represents the loss of information, is an important criterion to evaluate BBA approximations. An iterative approximation approach is proposed based on minimizing the distance, i.e., maximizing the closeness between the approximated BBA in current iteration and the BBA obtained in the previous iteration. The iteration stops when the desired number of focal elements is reached. Furthermore, as aforementioned, the available performance evaluations of BBA approximations often use the computational time and the closeness between the original BBA and the approximated one to judge the goodness of BBA approximation approaches. How to evaluate the performance of BBA approximations is still an open problem. Therefore, in this paper, besides the new BBA approximation approach, the evaluations of BBA approximations are briefly reviewed. Two new approaches of performance evaluation for BBA approximations are also proposed from new perspectives including the preservation of uncertainty order, the preservation of probabilistic decision making. Experimental results based on the comparisons with other approaches and related analyses show that our new BBA approximation is rational and effective.

The rest of this paper is organized as follows. Section 2 introduces the basics of the theory of belief functions including the problem of high computational cost encountered, especially in evidence combination. Section 3 provides a brief review of major existing works on BBA approximations. In Section 4, a new iterative BBA approximation approach is proposed based on the distance of evidence. Illustrative examples are provided. The tradeoff between tractability and the optimality is discussed and analyzed based on some experiment in Section 4. Section 5 further discuss on the performance evaluation for the BBA approximations, based on which, the experiments, simulations and related analyses are provided in Section 7. Section 7 concludes this paper.

## Basics of The Theory of Belief Functions

The theory of belief function was proposed by Dempster and then further developed by Shafer [1], therefore, it is also called Dempster-Shafer theory (DST) or evidence theory, which is attractive in the fields related to uncertainty modeling and reasoning. The frame of discernment (FOD) Θ = {*θ*_1_, …, *θ*_*n*_} is a basic concept in DST, where the elements are mutually exclusive and exhaustive. A basic belief assignment (BBA) over an FOD Θ is defined as

$$\sum_{A\subseteq \Theta }m(A)=1,\;m\left(\emptyset \right)=0$$ (1)

If *m*(*A*)>0 holds, *A* is called a focal element. Based on a BBA, the corresponding belief function and plausibility function are defined respectively as

$$Bel\left(A\right)=\sum_{B\subseteq A}m(B);\;Pl\left(A\right)=\sum_{A\cap B\neq \emptyset }m(B)$$ (2)

[*Bel*(*A*), *Pl*(*A*)] is called the belief interval of *A* representing the degree of imprecision for the focal element *A*.

Dempster’s rule of combination is for combining distinct pieces of evidence, which is defined as follows. ∀*A* ∈ 2^Θ^:

$$m\left(A\right)=\left\{\begin{array}{c} 0,\;\text{if}\;A=\emptyset \\ \frac{\sum_{A_{i}\cap B_{j}=A}m_{1}\left(A_{i}\right)m_{2}\left(B_{j}\right)}{1-K},\;\text{if}\;A\neq \emptyset \end{array}\right.$$ (3)

where *A*_*i*_ represents a focal element of *m*_1_; *B*_*j*_ represents a focal element of *m*_2_, and

$$K=\sum_{A_{i}\cap B_{j}=\emptyset }m_{1}\left(A_{i}\right)m_{2}\left(B_{j}\right)$$ (4)

is called the coefficient of conflict, which represents the total degree of conflict between pieces of evidence. Many alternative rules [5] were proposed to redistribute the conflict. Besides the evidence combination, there are also other operations based on BBAs in DST such as marginalization, conditioning, etc [1].

To describe the degree of closeness between two pieces of evidence, the definitions on distance of evidence are required. Jousselme’s distance *d*_*J*_[14] is one of the most commonly used one, which is defined as

$$d_{J}\left(m_{1},m_{2}\right)=\sqrt{0.5\cdot {\left(m_{1}-m_{2}\right)}^{T}\text{Jac}\;\left(m_{1}-m_{2}\right)}$$ (5)

Here, **Jac** is Jaccard’s weighting matrix and its elements **Jac**(*A*, *B*) for focal elements *A* and *B* are defined as

$$\text{Jac}\left(A,B\right)=\frac{\left|A\cap B\right|}{\left|A\cup B\right|}$$ (6)

Jousselme’s distance has been proved to be a strict distance metric [15].

DST has been argued due to its limitations [5]. One of the limitations is the high computational cost encountered in all kinds of operations including the evidence combination, conditioning, marginalization, and belief and plausibility degrees calculation in DST [6], especially when large amount of focal elements are available. In the next subsection, we illustrate the high computational cost problem by the example of evidence combination using the classical Dempster’s rule of combination.

### Computational cost analysis for evidence combination

Suppose that an FOD |Θ| = *n*. A BBA *m* is defined on Θ. Therefore, there are at most 2^*n*^ − 1 focal elements for *m*. Here we analyze the computational cost for the combination between *m* and itself according to Dempster’s rule in Eq (3) in terms of the multiplication operation included. There are at most (2^*n*^ − 1) × (2^*n*^ − 1) times of multiplication. Suppose that the actual number of focal elements (with non-zero mass assignments) for the BBA *m* is *s*. Then, there are *s* ⋅ *s* = *s*^2^ times of multiplication, because it is meaningless for those 2^*n*^ − 1 − *s* focal elements with zero mass assignments to attend the combination. Therefore, when there are too many focal elements, the computational cost will be high, which is harmful for the practical use of Dempster’s rule of combination [6]. If we can reduce the value of *s*, that is, to reduce the number of focal elements, the computational cost could be reduced. Therefore, various BBA approximation approaches [7–13] were proposed, which approximate the original BBA with a simpler one (having less focal elements), thus to reduce the computational cost of evidence combination.

Note that some researchers design efficient algorithms for evidence combination. The representatives of this type of approaches include [16, 17], and [18]. Besides the design of efficient combination algorithms, the BBA approximation is another way to reduce the computational cost, which is more intuitive for human to catch the meaning [19]. In this paper, we focus on the BBA approximations.

## Brief Review of BBA Approximations

The available BBA approximation approaches can be categorized into two types from the viewpoint of the technical implementation, i.e., to preset the number of remaining focal elements or to preset the maximum allowed cardinality of the remaining focal elements.

They can also be categorized into three major types depending on the criterion used for removing focal elements as shown in Table 1.

**Table 1:** Categorization of BBA approximations.

**Category**	**Characteristics**
1^st^ Criterion	Using the mass values of focal elements
2^nd^ Criterion	Using the cardinalities of focal elements
3^rd^ Criterion	Joint use of both cardinalities and mass values

Major available BBA approximations are briefly reviewed below.

### Existing BBA Approximations: Type I

The first type of BBA approximation approaches is according to the criterion of the focal element’s mass assignment, where the focal elements with smaller mass assignments are deemed unimportant, which are first removed. In this type of approaches, there is usually a presetting of the number of remaining focal elements *k*. The removal of focal elements is continued until the number of remaining focal elements reaches *k*. *k* − *l* − *x*[7], Summarization [8] and *D*_1_[9] are representatives for this type.

#### *k* − *l* − *x* method [7]

This classical approach was proposed by [7], which has three parameters. The approximated BBA is obtained by

1. keeping no less than *k* focal elements;
2. keeping no more than *l* focal elements;
3. deleting the masses which are no larger than *x*.

In *k* − *l* − *x* approach, all focal elements in the original BBA are sorted according to their mass assignments in a decreasing order. Then, the top *p* focal elements are selected so that *k* ≤ *p* ≤ *l* and so that the sum of mass assignments of these top *p* focal elements is no less than 1 − *x*. The removed mass assignments are then redistributed to those remaining focal elements by a classical normalization procedure. In fact, in *k* − *l* − *x* method, the focal elements with smaller mass assignments are regarded as “unimportant” ones and thus removed first.

#### Summarization method [8]

This method also keeps focal elements having largest mass values as in *k* − *l* − *x* method. The mass assignments of removed focal elements are accumulated and assigned to their union set. Suppose that *m* is the original BBA and *k* is the desired number of remaining focal elements in the approximated BBA *m*_*S*_. Let *M* denotes the set of *k* − 1 focal elements having the largest mass assignments in *m*(⋅). Then *m*_*S*_(⋅) is obtained from *m*(⋅) by

$$m_{S}\left(A\right)=\left\{\begin{array}{cc} m(A), & \;\;\;\;\text{if}\;A\in M\\ \sum_{A^{\prime }\subseteq A,A^{\prime }\notin M}m\left(A^{\prime }\right), & \;\;\;\;\text{if}\;A=A_{0}\\ 0, & \;\;\;\;\text{otherwise} \end{array}\right.$$ (7)

where *A*_0_ is

$$A_{0}\;≜\underset{A^{\prime }\notin M,m(A^{\prime })>0}{\cup }A^{\prime }$$ (8)

Note that the number of remaining focal elements could be *k* (if *A*_0_ ∉ *M*) or *k* − 1 (if *A*_0_ ∈ *M*) after applying Summarization method.

#### D1 method [9]

Suppose that *m* is the original BBA and *m*_*S*_ denotes the approximated BBA. The desired number of remaining focal elements is *k*. Let *M* be the set of *k* − 1 focal elements with the largest mass assignments in *m* and *M*^−^ be the set which includes all the other focal elements of the original BBA *m*. D1 method is to keep all members of *M* as the focal elements of *m*_*S*_ and to re-assign the mass assignments of the focal elements in *M*^−^ among those focal elements in *M* according to the following procedure.

For a focal element *A* ∈ *M*^−^, in *M*, find out all the supersets of *A* to construct a collection *M*_*A*_. If *M*_*A*_ is non-empty, *A*’s mass assignment is uniformly assigned among those focal elements having smallest cardinality in *M*_*A*_. When *M*_*A*_ is empty, then construct $M_{A}^{\prime }$ as

$$M_{A}^{\prime }=\left\{B\in M|\left|B\right|\geq \left|A\right|,B\cap A\neq \emptyset \right\}$$ (9)

Then, if $M_{A}^{\prime }$ is non-empty, *m*(*A*) is assigned among those focal elements having smallest cardinality in $M_{A}^{\prime }$. The value assigned to a focal element *B* depends on the cardinality of *B*∩*A*, i.e., |*B*∩*A*|. The above procedure is iteratively executed until all *m*(*A*) have been re-assigned to those focal elements in collection *M*.

If $M_{A}^{\prime }$ is empty, we have two possible cases:

1. If the univeral set Θ ∈ *M*, the sum of mass assignments of those focal elements in *M*^−^ will be added to Θ;
2. If Θ ∉ *M*, then let Θ be a focal element of *m*_*S*_(⋅) and assign the sum of mass assignments of those focal elements in *M*^−^ to *m*_*S*_(Θ).

Note that the number of remaining focal elements is *k* − 1, if Θ ∈ *M*. See [9] for more details of D1 method.

### Existing BBA Approximations: Type II

The second type of BBA approximation approaches are based on the criterion of the focal elements’ cardinality. The focal elements with larger cardinalities, which might cause more computational cost, are first removed. *k*-additive approximation [10], hierarchical proportional redistribution approach [20] are representatives of the 2nd type, where the maximum allowed cardinality of the remaining focal element is preset. See related reference for details.

### Existing BBA Approximations: Type III

The third type of BBA approximation approaches are based on the criterion of joint using the mass assignments and cardinality of focal elements to determine which focal element should be first removed. Denœux’s inner and outer approximations [11], and our previous works including the rank-level fusion based approximation [12], and the non-redundancy based BBA approximations [13] belong to this type. In these approaches, the number *k* of remaining focal elements is preset.

#### Denœux’s inner and outer approximations [11]

In Denœux’s inner and outer approximations, two different distances between focal elements (intersection-based *δ*_∩_ and union-based *δ*_∪_) are used, which are defined as

$$\begin{array}{cc} \delta_{\cup }\left(A_{i},A_{j}\right) & =\left[m\left(A_{i}\right)+m\left(A_{j}\right)\right]\cdot \left|A_{i}\cup A_{j}\right|\\ & -m\left(A_{i}\right)\cdot \left|A_{i}\right|-m\left(A_{j}\right)\cdot \left|A_{j}\right| \end{array}$$ (10)

$$\begin{array}{cc} \delta_{\cap }\left(A_{i},A_{j}\right) & =m\left(A_{i}\right)\cdot \left|A_{i}\right|+m\left(A_{j}\right)\cdot \left|A_{j}\right|\\ & -\left[m\left(A_{i}\right)+m\left(A_{j}\right)\right]\cdot \left|A_{i}\cap A_{j}\right| \end{array}$$ (11)

Such distances between focal elements consider both the information of the cardinality and mass assignment of the focal element.

“Similar” focal elements defined based on *δ*_∩_ are aggregated by the intersection operation to reduce the number of focal elements, which is called the inner approximation. “Similar” focal elements defined based on *δ*_∪_ are aggregated by the union operation to reduce the number of focal elements, which is called the outer approximation. The focal elements are pairwise aggregated till the desired focal elements number is reached. Note that the focal element obtained using aggregation (no matter union or intersection) might be a focal element of the original BBA, therefore, the number of remaining focal elements cannot be estimated precisely. Furthermore, inner approximation might bring the empty set ∅ using intersection operation, which is not allowed in the classical “closed-world” DST.

See [11] for more details and illustrative examples.

#### Rank-level fusion based approximation [12]

In rank-level fusion based approximations, two ranks of all focal elements in the original BBA are obtained based on each focal element’s cardinality and mass assignment, respectively. Then two ranks are fused to generate a new rank, which can describe both the information of mass assignment and the cardinality. The focal elements with smaller rank position will be removed at first, which represents that they are with smaller mass assignments and with bigger cardinality size at the same time. The procedure is briefly introduced below.

1. Sort all the focal elements of an original BBA (with *L* focal elements) according to the mass assignment values (in ascending order which is due to the assumption that the focal element with small mass should be deleted as early as possible). The rank vector obtained is
  $$r_{m}=\left[r_{m}\left(1\right),r_{m}\left(2\right),...,r_{m}\left(L\right)\right]$$ (12)
  where *r*_*m*_(*i*) denotes the rank position of the *i*th focal elements (*i* = 1, …, *L*) in the original BBA according to the mass assignment.
2. Sort all the focal elements of the original BBA according to the cardinalities (in descending order, this is due to the assumption that the focal element with big cardinality should be deleted as early as possible). The rank vector can be obtained as
  $$r_{c}=\left[r_{c}\left(1\right),r_{c}\left(2\right),...,r_{c}\left(L\right)\right]$$ (13)
  where *r*_*c*_(*i*) denotes the rank position of the *i*th focal elements in the original BBA according to the cardinality.
3. According to the rank-level fusion, we can obtain a fused rank as
  $$r_{f}=\left[r_{f}\left(1\right),r_{f}\left(2\right),...,r_{f}\left(L\right)\right]$$ (14)
  where
  $$r_{f}\left(i\right)=\alpha \cdot r_{f}\left(i\right)+\left(1-\alpha \right)\cdot r_{c}\left(i\right)$$ (15)
  and *α* ∈ [0, 1] is used to show the preference of two different criteria. Such a fused rank can be regarded as a more comprehensive criterion containing both the information of mass assignments and cardinality.
4. Sort *f*_*f*_ in ascending order and find the focal element with the smallest *r*_*f*_ value, i.e., $r_{f}\left(j\right)=\min_{i}rf\left(i\right)$. Then remove the *j*th focal element in the original BBA.
5. Repeat steps 1) -5) till only *k* focal elements remain. Do re-normalization of the remaining *k* focal elements. Finally, output the approximated BBA.

See [12] for more details and illustrative examples.

#### Non-redundancy based approximations [13]

In non-redundancy based approximations, the degrees of non-redundancy are defined based on the distances of focal elements as shown in Eq (11). First, calculate the distance matrix for all the focal elements of *m*(⋅) as

$$Mat_{FE}≜\left[\begin{array}{cccc} \delta_{\cap }\left(A_{1},A_{1}\right) & \delta_{\cap }\left(A_{1},A_{2}\right) & \cdots & \delta_{\cap }\left(A_{1},A_{l}\right)\\ \delta_{\cap }\left(A_{2},A_{1}\right) & \delta_{\cap }\left(A_{2},A_{2}\right) & \cdots & \delta_{\cap }\left(A_{2},A_{l}\right)\\ \vdots & \vdots & \ddots & \vdots \\ \delta_{\cap }\left(A_{l},A_{1}\right) & \delta_{\cap }\left(A_{l},A_{2}\right) & \cdots & \delta_{\cap }\left(A_{l},A_{l}\right) \end{array}\right]$$

It should be noted that *δ*_∩_(*A*_*i*_, *A*_*i*_) = 0 and *δ*_∩_(*A*_*i*_, *A*_*j*_) = *δ*_∩_(*A*_*j*_, *A*_*i*_) where *i* = 1, …, *l*. Hence, it is not necessary to calculate all the elements in *Mat*_*FE*_ because the matrix is symmetric.

We define the degree of non-redundancy of the focal element *A*_*i*_ by

$$\text{nRd}\left(A_{i}\right)≜\frac{1}{l-1}\sum_{j=1}^{l-1}\delta_{\cap }\left(A_{i},A_{j}\right)$$ (16)

As we can see that the definitions on degree of redundancy also use both the information of the cardinality and mass assignment of the focal element. The larger nRd(*A*_*i*_) value, the larger non-redundancy (less redundancy) for *A*_*i*_. The less nRd(*A*_*i*_) value, the less non-redundancy (larger redundancy) for *A*_*i*_. Those more redundant focal elements should be removed at first. See [13] for more details and illustrative examples.

The categorization, pros and cons of the above BBA approximations are illustrated in Table 2 below.

**Table 2:** Comparisons of different BBA approximations.

**BBA Approximations**	**Category**	**Advantages**	**Drawbacks**
*k* − *l* − *x*	1st	Simple and intuitive; One can select different parameters to control the number of remaining focal elements and the loss of information (maximum allowed mass values to remove)	The result depends on the parameter selection. The number of remaining focal elements cannot be estimated precisely
Summarization	1st	Same as *k* − *l* − *x*	Not accurate caused by its normalization step
D1	1st	Same as *k* − *l* − *x*	Relatively high computational cost
HPR	2nd	Hierarchical implementation; The maximum cardinality of the remaining focal elements can be controlled.	Relatively high computational cost; The number of remaining focal elements cannot be controlled.
Rank-level fusion	3rd	Use both the information of mass values and cardinalities of focal elements; The joint use is implemented by using simple yet effective rank-level fusion	The results depend on the weighs of the two criteria. The results depend on the rank fusion rule; High computational cost due to the double criteria used;
Inner approximations	3rd	Use both the information of mass values and cardinalities of focal elements; The similar or unimportant focal elements are replaced by their intersection.	The approximate BBA might have the empty set, which is not allowed in closed-world assumption; The strictness of definition of the similarity between focal elements needs further verification; High computational cost; The number of remaining focal elements cannot be estimated precisely
Outer approximations	3rd	Using the information of both mass values and cardinalities of focal elements; The similar or unimportant focal elements are replaced by their union.	The strictness of definition of the similarity between focal elements needs further verification; High computational cost; The number of remaining focal elements cannot be estimated precisely.
Non-redundancy based approximation	3rd	Using the information of both mass values and cardinalities of focal elements; The most redundant focal element is removed at first;	The strictness of definition of the redundancy of focal elements needs further verification; High computational cost.

All the available works referred above are rational and make sense to some extent. The approaches in the first and second types only emphasize one aspect of the information in a focal element. The approaches in the third type are more rational due to the joint use of the mass assignments and cardinalities. Although the available works in the third type are in better direction, the current joint use of mass assignments and cardinalities still have some limitations.

For example, in the rank-level based approximation [12], although it is simple, but it is at the price of loss of information due to rank-level fusion when compared with the data-level fusion. Furthermore, there exists the problem of parameter (the weights of mass assignment and cardinality) selection.

In Denœux’s inner and outer approximations [11], the strictness of the distances between focal elements are not seriously checked till now. Furthermore, when using inner approximation, the empty set ∅ can be generated as a focal element, which is not allowed in the classical DST based on the closed-world assumption.

Also in non-redundancy degree based approximations [13], the rationality and strictness of the definitions on non-redundancy for focal elements still need further inspections.

In summary, the current joint use of mass assignments and cardinalities for the BBA approximation is still an open problem. Therefore, in this paper, we propose another approximation according to the joint use of the mass assignment and the cardinality from a different angle, which is introduced in the next section.

## A Novel BBA Approximation Using Distance of Evidence

To be more direct and to use more information, in this paper, we start from the performance evaluation of BBA approximations for designing new BBA approximation approaches. The basic idea is as follows.

In the available related literatures, the performance evaluation of BBA approximations often includes the evaluation in terms of the computational cost and the evaluation in terms of the information loss.

Intuitively, a better BBA approximation should output a BBA having less computational cost for the operations in DST, e.g., the evidence combination, and being more similar to the original BBA (less loss of information). The similarity can be described using the distance of evidence in Eq (5). So far as we remove some focal elements, the computational cost will be decreased more or less. Here, we focus on the closeness between the approximated BBA and the original one.

In all possible approximated BBAs, the one having smaller distance from the original one is preferred. If one want to obtain such a BBA denoted by *m*_*opt*_, one can pick the one minimizing the chosen distance with respect to the original BBA among all the possible BBAs having *k* focal elements according to

$$\begin{array}{c} m_{opt}=m_{s}\\ s=\underset{i}{\arg}\min d_{J}\left(m_{i},m\right)\\ s.t.\left\{\begin{array}{c} i=1,...L\\ \text{the}\;\text{number}\;\text{of}\;\text{focal}\;\text{elements}\;\text{in}\;m_{i}\;\text{is}\;k \end{array}\right. \end{array}$$ (17)

However, such an optimal way is time-consuming and might cause the BBA approximation intractable, especially when the number of focal elements is very large (we provide a detailed analysis on this at the end of this section). Therefore, we try to make a trade-off between the optimality and the tractability by introducing an iterative implementation. We design an iterative BBA approximation approach based on minimizing the distance of evidence between the approximated BBA in current iteration and the BBA obtained in the previous iteration.

Given a BBA *m* with *L* focal elements *A*_1_, …, *A*_*L*_. Suppose that the desired number of remaining focal elements is *k*. The specific implementation is as follows.

**Step 1:** Remove a focal element *A*_*i*_ from *m*. Normalize the mass of the remaining *A*_*j*_, where *j* ≠ *i* and *j* ∈ {1, …, *L*}, to generate a new BBA $m_{i}^{\prime }$.
**Step 2:** Calculate the distance between $m_{i}^{\prime }$ and *m* denoted by *d*(*i*).
  Execute Step 1 and Step 2 for all *i* = 1, …, *L*.
**Step 3:** Find the minimum values of *d*(*i*), *i* = 1, …, *L*, i.e.,
  $$s=\underset{i}{\arg}\min d\left(i\right)$$
**Step 4:** Remove the focal element *A*_*s*_ from *m* and de the normalization to generate an approximated BBA *m*_*s*_. Such a new BBA *m*_*s*_ is closest to the BBA *m* compared with those obtained by removing any other focal element *A*_*j*_, where *j* ≠ *s*, *j* ∈ {1, …, *L*}. Reduce the number of focal elements by one, i.e., *L* = *L* − 1.
**Step 5:** Assign *m* = *m*_*s*_. If the desired number of remaining focal elements is not reached, i.e., *L* > *K*, then goto Step 1. If the desired number of remaining focal elements is reached, then output *m* as the final approximated BBA.

**Fig 1:**
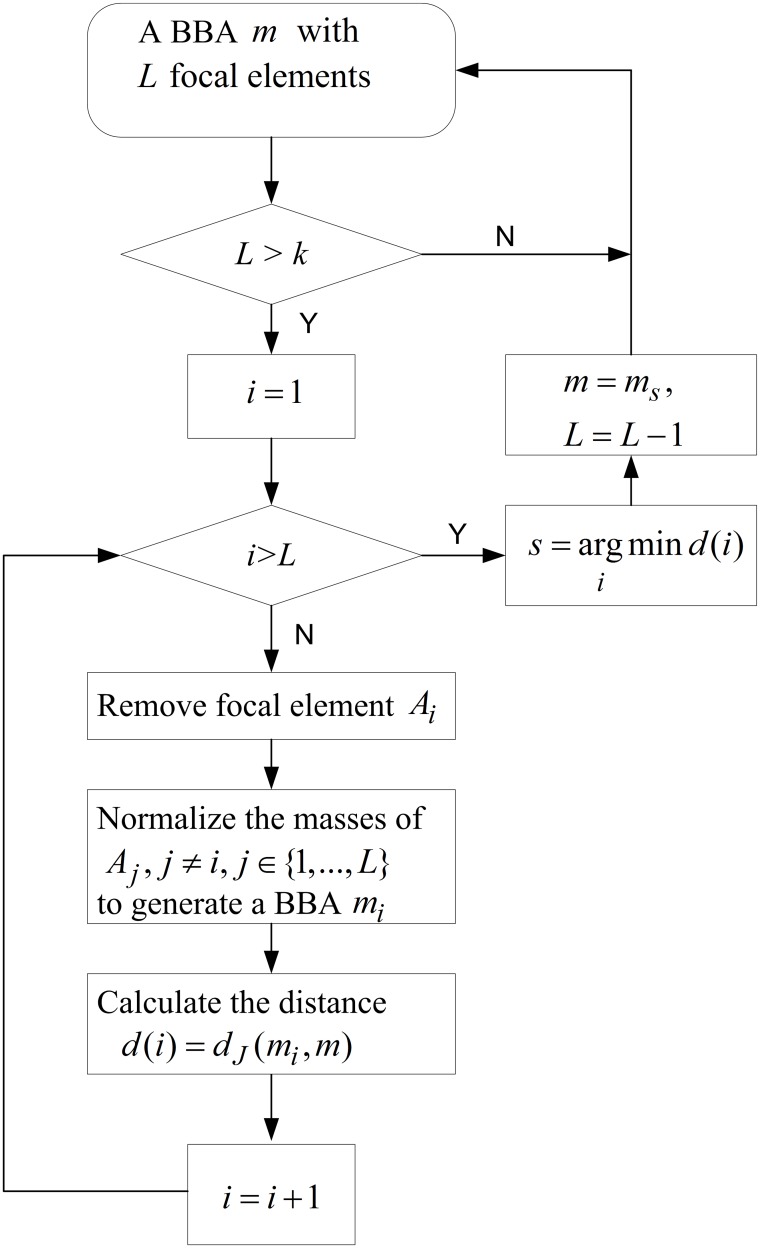
Procedure of the iterative BBA approximation using distance of evidence. Illustration of the whole procedure of the new iterative approximation.

The whole procedure is illustrated in Fig 1.

Here we provide an illustrative example to show how the BBA approximation using distance of evidence works.

### Example 1

Let’s consider the BBA *m*(⋅) defined over the FOD Θ = {*θ*_1_, *θ*_2_, *θ*_3_, *θ*_4_, *θ*_5_} listed in Table 3.

**Table 3:** Focal elements and mass values of *m*(⋅).

**Focal Elements**	**Mass assignments**
*A*_1_ = {*θ*_1_, *θ*_2_}	0.60
*A*_2_ = {*θ*_1_, *θ*_3_, *θ*_4_}	0.20
*A*_3_ = {*θ*_3_}	0.10
*A*_4_ = {*θ*_3_, *θ*_4_}	0.05
*A*_5_ = {*θ*_4_, *θ*_5_}	0.05

Here the number *k* of the remaining focal elements is set to 3. Now, we apply the iteration BBA approximation based on distance of evidence to *m*.

In the first iteration, when we remove *A*_1_ = {*θ*_1_, *θ*_2_}, the BBA obtained is as follows.

$$m_{1}\left(A_{2}\right)=0.5000,m_{1}\left(A_{3}\right)=0.2500,m_{1}\left(A_{4}\right)=0.1250,m_{1}\left(A_{5}\right)=0.1250.$$

The corresponding distance of evidence *d*_*J*_(*m*_1_, *m*) = 0.4899

When we remove *A*_2_ = {*θ*_1_, *θ*_3_, *θ*_4_}, the BBA obtained is

$$m_{2}\left(A_{1}\right)=0.7500,m_{2}\left(A_{3}\right)=0.1250,m_{2}\left(A_{4}\right)=0.0625,m_{2}\left(A_{5}\right)=0.6250.$$

and *d*_*J*_(*m*_2_, *m*) = 0.1431.

When we remove *A*_3_ = {*θ*_3_}, the BBA obtained is

$$m_{3}\left(A_{1}\right)=0.6667,m_{3}\left(A_{2}\right)=0.2222,m_{3}\left(A_{4}\right)=0.0556,m_{3}\left(A_{5}\right)=0.0556.$$

and *d*_*J*_(*m*_3_, *m*) = 0.0835

When we remove *A*_4_ = {*θ*_3_, *θ*_4_}, the BBA obtained is

$$m_{4}\left(A_{1}\right)=0.6316,m_{4}\left(A_{2}\right)=0.2105,m_{4}\left(A_{3}\right)=0.1053,m_{4}\left(A_{5}\right)=0.0526.$$

and *d*_*J*_(*m*_4_, *m*) = 0.0375

When we remove *A*_5_ = {*θ*_4_, *θ*_5_}, the BBA obtained is

$$m_{5}\left(A_{1}\right)=0.6316,m_{5}\left(A_{2}\right)=0.2105,m_{5}\left(A_{3}\right)=0.1053,m_{5}\left(A_{4}\right)=0.0526.$$

and *d*_*J*_(*m*_5_, *m*) = 0.0421.

*d*_*J*_(*m*_4_, *m*) is the smallest, therefore, the focal element removed in 1st iteration is *A*_4_. After the 1st iteration, the number of focal elements is 4.

Then, in the second iteration, the BBA to approximate is *mm* ← *m*_4_, i.e.,

$$mm\left(B_{1}\right)=0.6316,mm\left(B_{2}\right)=0.2105,mm\left(B_{3}\right)=0.1053,mm\left(B_{4}\right)=0.0526.$$

where *B*_1_ = *A*_1_, *B*_2_ = *A*_2_, *B*_3_ = *A*_3_, *B*_4_ = *A*_5_.

When we remove *B*_1_ = {*θ*_1_, *θ*_2_}, the BBA obtained is

$$mm_{1}\left(B_{2}\right)=0.5714,mm_{1}\left(B_{3}\right)=0.2857,mm_{1}\left(B_{4}\right)=0.1429.$$

and *d*_*J*_(*mm*_1_, *mm*) = 0.5077.

When we remove *B*_2_ = {*θ*_1_, *θ*_3_, *θ*_4_}, the BBA obtained is

$$mm_{2}\left(B_{1}\right)=0.8000,mm_{2}\left(B_{3}\right)=0.1333,mm_{2}\left(B_{4}\right)=0.0667.$$

and *d*_*J*_(*mm*_2_, *mm*) = 0.1589.

When we remove *B*_3_ = {*θ*_3_}, the BBA obtained is

$$mm_{3}\left(B_{1}\right)=0.7059,mm_{3}\left(B_{2}\right)=0.2353,mm_{3}\left(B_{4}\right)=0.0588.$$

and *d*_*J*_(*mm*_3_, *mm*) = 0.0909.

When we remove *B*_4_ = {*θ*_4_, *θ*_5_}, the BBA obtained is

$$mm_{4}\left(B_{1}\right)=0.6667,mm_{4}\left(B_{2}\right)=0.2222,mm_{4}\left(B_{3}\right)=0.1111.$$

and *d*_*J*_(*mm*_4_, *mm*) = 0.0454.

*d*_*J*_(*mm*_4_, *mm*) is the smallest, therefore, the focal element removed in 2nd iteration is *B*_4_, i.e., *A*_5_. After the 2nd iteration, the number of focal elements reaches 3 and then the iteration stops. The final output approximated BBA is *mo* ← *mm*_4_.

Here, we also provide the approximation results of the available BBA approximations referred in the previous section for comparisons.

#### Using *k* − *l* − *x* method [7]

Here *k* and *l* are set to 3. *x* is set to 0.1. The focal elements *A*_4_ = {*θ*_3_, *θ*_4_} and *A*_5_ = {*θ*_4_, *θ*_5_} are removed without violating the constraints in *k* − *l* − *x*. The remaining total mass value is 1 − 0.05 − 0.05 = 0.9. Then, all the remaining focal elements’ mass values are divided by 0.9 to accomplish the normalization. The approximated BBA $m_{S}^{klx}\left(\cdot \right)$ obtained by *k* − *l* − *x* method is listed in Table 4, where $A_{i}^{\prime }$, *i* = 1, 2, 3 are the focal elements of $m_{S}^{klx}\left(\cdot \right)$.

**Table 4:** $m_{S}^{klx}\left(\cdot \right)$ obtained using *k* − *l* − *x*.

Focal Elements	Mass values
$A_{1}^{\prime }=\{\theta_{1},\theta_{2}\}$	0.6667
$A_{2}^{\prime }=\{\theta_{1},\theta_{3},\theta_{4}\}$	0.2222
$A_{3}^{\prime }=\{\theta_{3}\}$	0.1111

#### Using summarization method [8]

Here *k* is set to 3. According to the summarization method, the focal elements *A*_3_ = {*θ*_3_}, *A*_4_ = {*θ*_3_, *θ*_4_} and *A*_5_ = {*θ*_4_, *θ*_5_} are removed, and their union {*θ*_3_, *θ*_4_, *θ*_5_} is generated as a new focal element with mass value *m*({*θ*_3_}) + *m*({*θ*_3_, *θ*_4_}) + *m*({*θ*_4_, *θ*_5_}) = 0.2. The approximated BBA $m_{S}^{Sum}$ is listed in Table 5 below.

**Table 5:** $m_{S}^{Sum}\left(\cdot \right)$ obtained using Summarization.

Focal Elements	Mass values
$A_{1}^{\prime }=\{\theta_{1},\theta_{2}\}$	0.60
$A_{2}^{\prime }=\{\theta_{1},\theta_{3},\theta_{4}\}$	0.20
$A_{3}^{\prime }=\{\theta_{3},\theta_{4},\theta_{5}\}$	0.20

#### Using D1 method [9]

Here *k* is still 3. It can be obtained that *A*_1_, *A*_2_ belong to *M*, and *A*_3_, *A*_4_, *A*_5_ belong to *M*^−^. The focal element *A*_1_ = {*θ*_1_, *θ*_2_} has empty intersection with the focal elements in *M*^−^, therefore its value will be unchanged. In *M*, *A*_2_ is the unique superset of *A*_3_ and *A*_4_, therefore, *m*(*A*_3_) + *m*(*A*_4_) = 0.10 + 0.05 is added to its original mass value. *A*_2_ also covers half of *A*_5_, therefore, *m*(*A*_5_)/2 = 0.025 is further added to the mass of *A*_2_. Finally, the rest mass value is assigned to the total set Θ. The approximated BBA $m_{S}^{D1}$ is listed in Table 6.

**Table 6:** $m_{S}^{D1}\left(\cdot \right)$ obtained using D1.

Focal Elements	Mass values
$A_{1}^{\prime }=\{\theta_{1},\theta_{2}\}$	0.60
$A_{2}^{\prime }=\{\theta_{1},\theta_{3},\theta_{4}\}$	0.375
$A_{3}^{\prime }=\Theta$	0.025

#### Using Rank-level fusion based approximation [12]

The number of remaining focal elements is 3. The approximate BBA is as shown in Table 7. It should be noted that although for Example 1, $m_{S}^{Rank}\left(\cdot \right)=m_{S}^{klx}\left(\cdot \right)$, they are two different approaches.

**Table 7:** $m_{S}^{Rank}\left(\cdot \right)$ obtained using Rank-level fusion.

Focal Elements	Mass values
$A_{1}^{\prime }=\{\theta_{1},\theta_{2}\}$	0.6667
$A_{2}^{\prime }=\{\theta_{1},\theta_{3},\theta_{4}\}$	0.2222
$A_{3}^{\prime }=\{\theta_{3}\}$	0.1111

#### Using Denœux inner and outer approximation [11]

With the inner approximation method, the focal elements pair with smallest Denouex’s inner distance are removed, and then their intersection is set as the supplemented focal element whose mass value is the sum of the removed two focal elements’ mass values. Such a procedure is repeated until the desired number of focal elements is reached. The results at each step are listed in Table 8.

**Table 8:** $m_{S}^{Rank}\left(\cdot \right)$ obtained using inner approximation.

Focal Elements	Mass values
$A_{1}^{\prime }=\{\theta_{1},\theta_{2}\}$	0.6000
$A_{2}^{\prime }=\{\theta_{1},\theta_{3},\theta_{4}\}$	0.2000
$A_{3}^{\prime }=\emptyset$	0.1111

As we can see in Table 8, it generates the empty set as a focal element, which is not allowed in the classical Dempster-Shafer evidence theory under close-world assumption.

With the outer approximation method, the focal elements pair with smallest Denouex’s outer distance are removed, and then their union is set as the supplemented focal element whose mass value is the sum of the removed two focal elements’ mass values. Such a procedure is repeated until the desired number of focal elements is reached. The results at each step are listed in Table 9.

**Table 9:** $m_{S}^{Rank}\left(\cdot \right)$ obtained using outer approximation.

Focal Elements	Mass values
$A_{1}^{\prime }=\{\theta_{1},\theta_{2}\}$	0.6000
$A_{2}^{\prime }=\{\theta_{1},\theta_{3},\theta_{4}\}$	0.3500
$A_{3}^{\prime }=\{\theta_{4},\theta_{5}\}$	0.0500

#### Using the redundancy-based batch approximation method [13]

The desired remaining focal element is set to *k* = 3. We first calculate the distance matrix *Mat*_*FE*_, based on which, the degree of non-redundancy for each focal elements of *m*(⋅) can be obtained. It is listed in Table 10.

**Table 10:** Non-redundancy for different focal elements.

Focal Elements	Mass values	nRd(*A*_*i*_)
*A*_1_ = {*θ*_1_, *θ*_2_}	0.60	1.2250
*A*_2_ = {*θ*_1_, *θ*_3_, *θ*_4_}	0.20	0.5125
*A*_3_ = {*θ*_3_}	0.10	0.4875
*A*_4_ = {*θ*_3_, *θ*_4_}	0.05	0.4125
*A*_5_ = {*θ*_4_, *θ*_5_}	0.05	0.5125

Since *A*_3_ and *A*_4_ at the bottom have the two least nRd values, they correspond the two focal elements with the lowest non-redundancy, i.e., the highest redundancy. Therefore, they are removed and their mass values are redistributed thanks to the classical normalization step. The approximated BBA $m_{S}^{BRd}$ is listed in Table 11.

**Table 11:** $m_{S}^{BRd}\left(\cdot \right)$ obtained using the batch approximation based on redundancy.

Focal Elements	Mass values
$A_{1}^{\prime }=\{\theta_{1},\theta_{2}\}$	0.7059
$A_{2}^{\prime }=\{\theta_{1},\theta_{3},\theta_{4}\}$	0.2353
$A_{3}^{\prime }=\{\theta_{4},\theta_{5}\}$	0.0588

### Further discussion on the iterative approximation based on distance of evidence

As aforementioned, the BBA obtained using the above iterative approximation is usually not the BBA which is closest to the original BBA given a desired number *k* of remaining focal elements, however it can significantly reduce the computational cost caused by the optimization, thus, is more tractable. It can also obtain relative smaller distance when compared with other BBA approximations as verified in the experiment below.

If we are too greedy for obtaining the minimum distance, the price is the significantly increased computational cost in BBA approximation. Given the number of remaining focal elements *k*, there are totally $C_{2^{n}-1}^{k}$ different BBAs. The approximated BBA with minimum distance to the original one should be selected out of the $C_{n}^{k}$ different BBAs. Using our iterative approximation, there are totally

$$\begin{array}{c} C_{2^{n}-1}^{1}+C_{2^{n}-1-1}^{1}+\cdots +C_{k+1}^{1}\\ =C_{2^{n}-1}^{1}+C_{2^{n}-1-1}^{1}+\cdots +C_{2^{n}-1-\left(2^{n}-1-\left(k+1\right)\right)}^{1}\\ =2^{n}-1+2^{n}-1-1+\cdots +2^{n}-1-\left(2^{n}-1-\left(k+1\right)\right)\\ =\left(2^{n}-1-k\right)\cdot \left(2^{n}-1\right)-\frac{\left(2^{n}-1-k\right)\left(2^{n}-1-k-1\right)}{2}\\ =2^{n}-2^{n-1}+k/2 \end{array}$$

BBAs. When the |Θ| = 5, the size of searching space for the one finding the minimum one and our iterative approximation are compared at different *k* as shown in Table 12.

**Table 12:** Size of searching space.

**Number of Remaining FEs**	**Optimal**	**Iterative**
30	31	31
29	465	61
28	4495	90
27	31465	118
26	169911	145
25	736281	171
24	2629575	196
23	7888725	220
22	20160075	243
21	44352165	265
20	84672315	286
19	141120525	306
18	206253075	325
17	265182525	343
16	300540195	360
15	300540195	376
14	265182525	391
13	206253075	405
12	141120525	418
11	84672315	430
10	44352165	441
9	20160075	451
8	7888725	460
7	2629575	468
6	736281	475
5	169911	481
4	31465	486
3	4495	490
2	465	493

**Fig 2:**
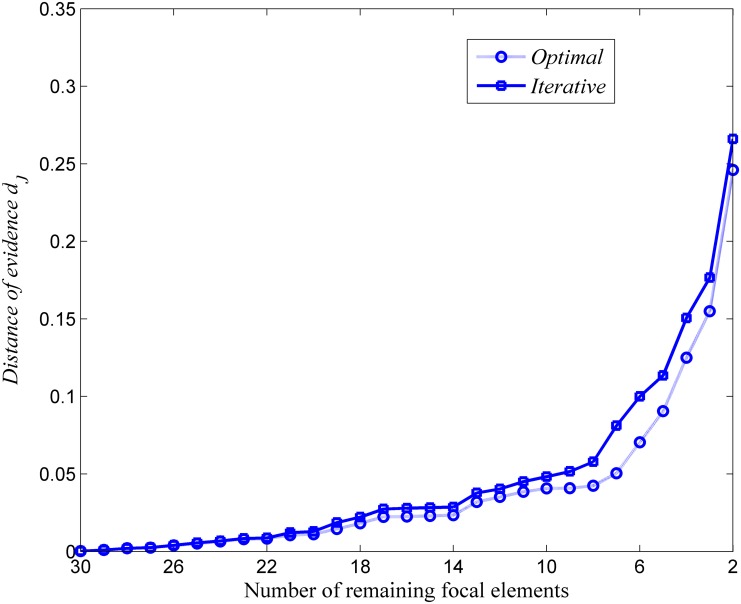
Comparisons on closeness for the optimal and the iterative approaches. Evaluation in terms of the loss of information for the optimal and iterative ways.

The distance of evidence between the approximated BBA and the original one for the one finding the minimum one and our iterative approximation are compared at different *k* as shown in Fig 2.

As shown in Fig 2 and Table 12, the approach to finding the minimum distance does provide the minimum loss of information, however, it is at the price of extremely large computational cost for the approximation, which makes the approximation intractable, especially when the |Θ| is very large. Our new proposed iterative approximation can make a good trade-off between the precision (with less loss of information) and the tractability (without so large computational cost).

To well justify our newly proposed BBA approximation, we give more detailed analyses on the evaluation of BBA approximations at first in the next section.

## Further analyses on evaluations of BBA approximations

### Evaluation criteria of BBA approximations

The evaluation criteria are very crucial for evaluate different BBA approximations, and also for design new BBA approximations. The available BBA approximation evaluation approaches are as follows.

**1) Reduction of the computational cost after approximation**

After the BBA approximation, the computational operations like evidence combination, conditioning, marginalization, belief and plausibility degrees evaluation will be reduced. The BBA approximation with larger degree of reduction is preferred.

**2) Closeness between the approximated BBA and the original one**

After the BBA approximation, the BBA obtained *m*′ will depart from the original one. Larger degree of such a departure, i.e., less closeness between the approximated BBA *m*′ and the original one *m*, means larger loss of information, which is not preferred. Such a degree of departure (or degree of closeness) can be described using the distance of evidence defined in Eq (5), i.e., *d*_*J*_(*m*, *m*′).

**3) Degree of ordering preservation between plausibilities of events**

In a BBA, each focal element corresponds to an event. Based on a given BBA *m*, we can calculate the corresponding plausibility function *Pl* according to Eq (2). Then, the ordering or ranking of the plausibilities *Pl*(*A*_*i*_), *A*_*i*_ ∈ 2^Θ^ of different focal elements (events) can be obtained, which is noted by Λ_*Pl*_. After the BBA approximation, we obtain the approximated BBA *m*′ and can calculate its plausibilities *Pl*′(*B*_*i*_), *B*_*i*_ ∈ 2^Θ^ and its ordering denoted by Λ_*Pl*′_. We can calculate the distance between Λ_*Pl*_ and Λ_*Pl*′_ using Spearman’s distance [21]:

$$d_{Pl}=1-\frac{\left(\Lambda_{\text{Pl}}-{\bar{\Lambda }}_{\text{Pl}}\right){\left(\Lambda_{{\text{Pl}}^{\prime }}-{\bar{\Lambda }}_{{\text{Pl}}^{\prime }}\right)}^{T}}{\sqrt{\left(\Lambda_{\text{Pl}}-{\bar{\Lambda }}_{\text{Pl}}\right){\left(\Lambda_{\text{Pl}}-{\bar{\Lambda }}_{\text{Pl}}\right)}^{T}}\cdot \sqrt{\left(\Lambda_{{\text{Pl}}^{\prime }}-{\bar{\Lambda }}_{{\text{Pl}}^{\prime }}\right){\left(\Lambda_{{\text{Pl}}^{\prime }}-{\bar{\Lambda }}_{{\text{Pl}}^{\prime }}\right)}^{T}}}$$ (18)

where smaller distance representing less loss of information or less distortion brought by the approximation is preferred.

**4) Preservation of inclusion relation**

Suppose that two BBAs *m*_1_ and *m*_2_ satisfy the inclusion relation (e.g., *s*-inclusion), which is defined as follows [22].

Suppose that *m*_1_’s focal elements are {*A*_1_, …, *A*_*q*_} and *m*_2_’s focal elements are {*B*_1_, …, *B*_*p*_}. If and only if there exists a non-negative matrix *G* = [*g*_*i*,*j*_] such that for *j* = 1, …, *p*, $\sum_{i=1}^{q}g_{ij}=1,g_{ij}>0\Rightarrow A_{i}\subseteq B_{j}$, and for *i* = 1, …, *q*, $\sum_{j=1}^{p}m_{2}\left(B_{j}\right)g_{i,j}=1$, where *g*_*ij*_ is the proportion of *B*_*j*_ that “flows down” to *A*_*i*_. That is, *m*_1_ is *s*-included in *m*_2_ (*m*_1_⊑_*s*_ *m*_2_) if the mass of any focal element *B*_*j*_ of *m*_2_ can be redistributed among subsets of *B*_*j*_ in *m*_1_. This means that *m*_1_ is less informative than *m*_2_.

After the approximation, if their approximated BBAs $m_{1}^{\prime }$ and $m_{2}^{\prime }$ still satisfy the inclusion relation, such a BBA approximation is preferred. This represents the informative relation between two BBAs is not affected by the approximation.

It should be noted that preservation of inclusion relation is a very strict relation. We have checked that all the BBA approximations introduced in this paper including our new approach cannot satisfy it.

Besides the above criteria, in this paper, we propose two new evaluation criteria for BBA approximations as follows.

**New criterion I: Order preservation in terms of uncertainty degrees**

Given *t* different BBAs (according to Algorithm 1 [23] in Table 2): *m*_1_, …, *m*_*t*_ and calculate their corresponding degree of uncertainty, e.g., the aggregated uncertainty (AU) [24, 25] as defined below.

$$\text{AU}\left(m\right)=\max_{P_{m}}\left[-\sum_{\theta \in \Theta }p_{\theta }\log_{2}p_{\theta }\right]$$ (19)

where the maximum is taken over all probability distributions being consistent with the given BBA. $P_{m}$ consists of all probability distributions 〈*p*_*θ*_|*θ*∈Θ〉 satisfying:

$$\left\{\begin{array}{c} p_{\theta }\in \left[0,1\right],\forall \theta \in \Theta \\ \sum_{\theta \in \Theta }p_{\theta }=1\\ Bel\left(A\right)\leq \sum_{\theta \in A}p_{\theta }\leq 1-Bel\left(\bar{A}\right),\;\forall A\subseteq \Theta \end{array}\right.\;$$ (20)

For the *t* BBAs, their corresponding AU values are *AU*(*m*_1_), …, *AU*(*m*_*t*_). Then, sort AU values in an ascending order to obtain a ranking Λ_*o*_. Apply a BBA approximation approach *S*_*i*_ to all the *t* BBAs, then *t* approximated BBAs can be obtained as $m_{1}^{i},...,m_{t}^{i}$. Calculate and sort the AU values also in an ascending order to obtain a ranking Λ_*i*_. Calculate the distance between Λ_*o*_ and Λ_*i*_.

If two rankings before and after the approximation *S*_*i*_ are closer to each other, then *S*_*i*_ is preferred for such an capability of order preservation, which represents a less loss of information or less distortion of the relation between BBAs.

Here, we can use the Spearman’s distance [21] to measure the rankings’ difference as follows.

$$d_{o,i}=1-\frac{\left(\Lambda_{o}-{\bar{\Lambda }}_{o}\right){\left(\Lambda_{i}-{\bar{\Lambda }}_{i}\right)}^{T}}{\sqrt{\left(\Lambda_{o}-{\bar{\Lambda }}_{o}\right){\left(\Lambda_{o}-{\bar{\Lambda }}_{o}\right)}^{T}}\cdot \sqrt{\left(\Lambda_{i}-{\bar{\Lambda }}_{i}\right){\left(\Lambda_{i}-{\bar{\Lambda }}_{i}\right)}^{T}}}$$ (21)

**New criterion II: Preservation of probabilistic decision**

After we apply the Pignisitc Probability Transformation (PPT) [26] to a BBA *m* according to

$$\text{BetP}\left(\theta_{i}\right)=\sum_{\theta_{i}\in B,\;B\in 2^{\Theta }}\frac{m(B)}{\left|B\right|},$$ (22)

the decision can be made by selecting the *θ*_*i*_ with the maximum value in BetP(*θ*_*j*_), *j* = 1, …, |Θ|.

If the probabilistic decision for the approximated BBA *m*′ (using *S*_*i*_) is the same as the probabilistic decision obtained based on the original BBA *m*, then the approximation *S*_*i*_ is desired.

Such a criterion describes the probabilistic decision consistency before and after the BBA approximation.

One can use Monte Carlo method based on all the above criteria to evaluate BBA approximations. See details of implementation in the simulations in the next section.

### Experiments and Simulations

In this section, we compare all the BBA approximation approaches aforementioned with the preset of remaining focal elements’ number *k* including *k* − *l* − *x* (denoted by *S*_1_), Summarization (*S*_2_), D1 (*S*_3_), rank-level fusion based approximation (*S*_4_), the non-redundancy based approximation (*S*_5_), and Denœux’s outer approximations (*S*_6_) (we do not compare inner approximation method which might bring troubles for making the comparisons because Jousselme’s distance cannot be computed if one allows to assign positive mass on empty set due to |∅| = 0), with our newly proposed BBA approximation approach (*S*_7_).

#### Simulation I—Comparisons in terms of computational time and the closeness

**Fig 3:**
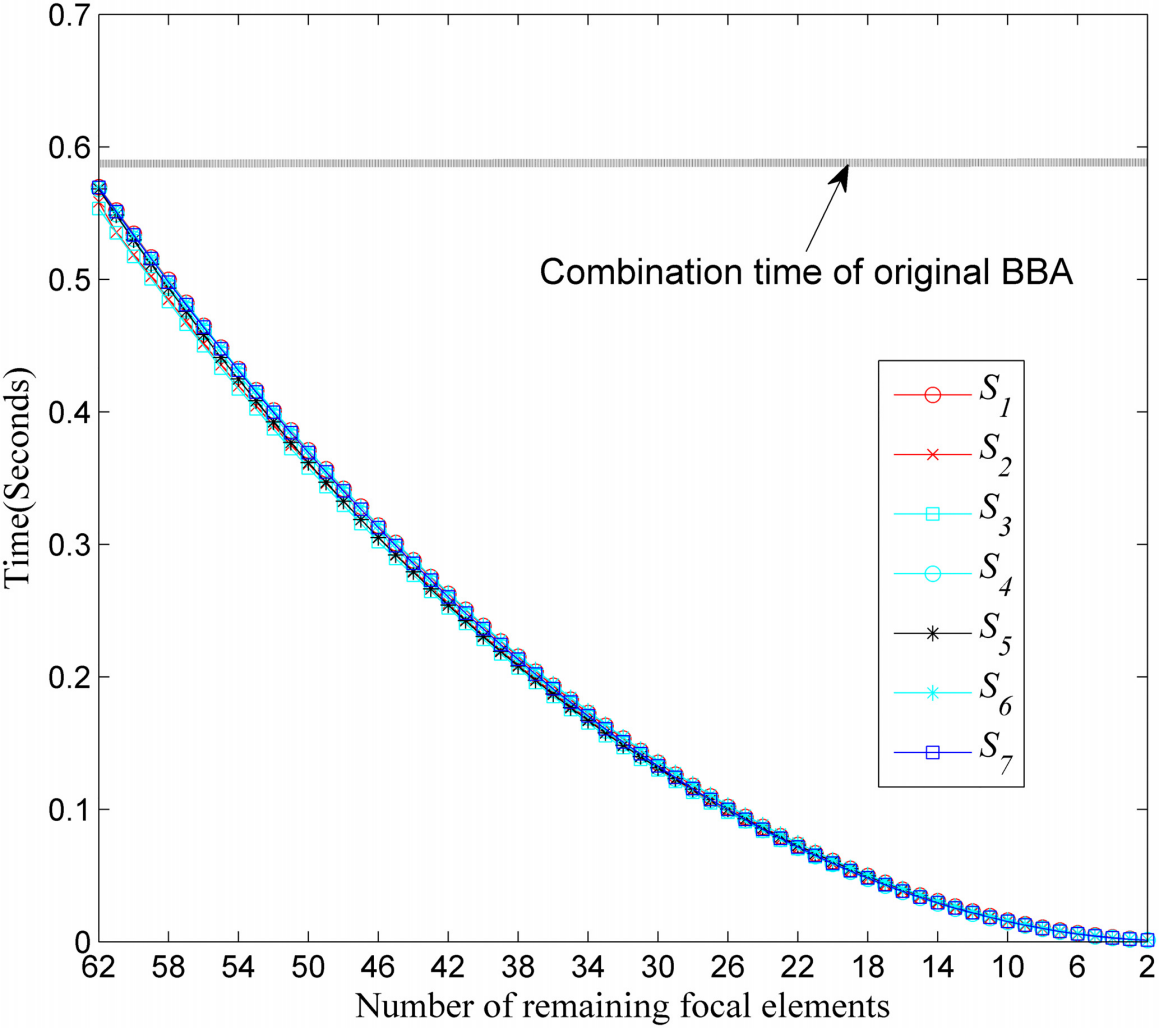
Computation time comparisons. Evaluation in terms of computational cost.

**Fig 4:**
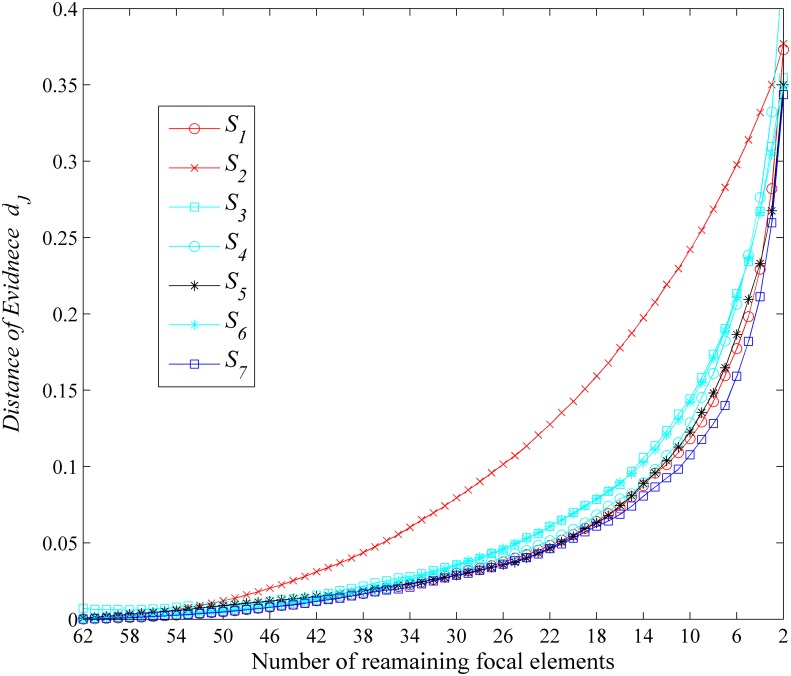
Closeness comparisons. Evaluation in terms of the loss of information.

In Simulation I, we use the computational time of the evidence combination and the distance of evidence between the approximated BBA and the original one in average as performance measures. Our comparative analysis is based on a Monte Carlo simulation using *M* = 200 random runs. The size of the FOD is |Θ| = 6. In the *j*th simulation run, a BBA *m*^*j*^(⋅) is randomly generated according to Algorithm 1 [23] in Table 13. In each random generation, there are 2^6^ − 1 = 63 focal elements in the BBA *m*^*j*^(⋅) to approximate. The number of remaining focal elements *k* for all the approaches used here are set to from 62 to 2. Then, different approximation results $\left\{m_{i,k}^{j}\left(\cdot \right)\right\}$ in the *j*th run can be obtained using the different approximation *S*_*i*_ (*i* represents the *i*th BBA approximation approach applied) given different remaining number (*k*) of focal elements. We record the computational time of the original evidence combination of *m*^*j*^(⋅)⊕*m*^*j*^(⋅) with Dempster’s rule of combination, and the computation time of using the Dempster’s rule of combination for each approximated BBA $m_{i,k}^{j}\left(\cdot \right)⊕m_{i,k}^{j}\left(\cdot \right)$. The average (over 200 runs) computation time for the original combination and the combination after the approximation are shown in Fig 3. The average (over 200 runs) distance values (*d*_*J*_) between the original BBA and the approximated BBA’s obtained using different approaches given different remaining focal elements’ number (*k*) are shown in Fig 4.

**Table 13:** Algorithm 1: Random BBA generation—Uniform sampling from all focal elements.

**Input**: Θ: Frame of discernment;
*N*_*max*_: Maximum number of focal elements
**Output**: **Output**: *m*: BBA
Generate P(Θ), which is the power set of Θ;
**FOReach** 1≤i≤|P(Θ)| do
Generate a value according to the Gamma distribution $G\left(1,1\right)\to m_{i}$,
**END**
Normalize the vector $m=\left[m_{1},...,m_{\left|P(\Theta )\right|}\right]\to m^{\prime }$;
$m\left(A_{i}\right)=m_{i}^{\prime }$;

As we can see, when compared with the original computational time, the computation time of all the BBA approximation approaches can be reduced. This is intuitive, because the number of focal elements are reduced. At the same time, our newly proposed approximation usually has the smallest distance of evidence, i.e., the largest closeness, which represents the least loss of information when compared with other approximations concerned here.

#### Simulation II—Comparisons in terms of order-preservation for uncertainty degree

In Simulation II, we compare the information preservation capability of different BBA approximations from another aspect. Suppose that the size of FOD is 5.

1. Randomly generate 5 different BBAs (according to Algorithm 1 in Table 2): *m*_1_, …, *m*_5_ and calculate their corresponding AU *AU*(*m*_1_), …, *AU*(*m*_5_).
2. Sort AU values in an ascending order to obtain a rank Λ_*o*_.
3. Apply a BBA approximation approach *S*_*i*_ to all the five BBAs, then five approximated BBAs can be obtained as $m_{1}^{i},...,m_{5}^{i}$.
4. Calculate and sort the AU values also in an ascending order to obtain a rank Λ_*i*_.
5. Calculate the distance between Λ_*o*_ and Λ_*i*_.

If two rankings before and after the approximation *S*_*i*_ are closer to each other, then *S*_*i*_ is preferred for such an capability of order preservation, which represents a less loss of information from another aspect.

**Fig 5:**
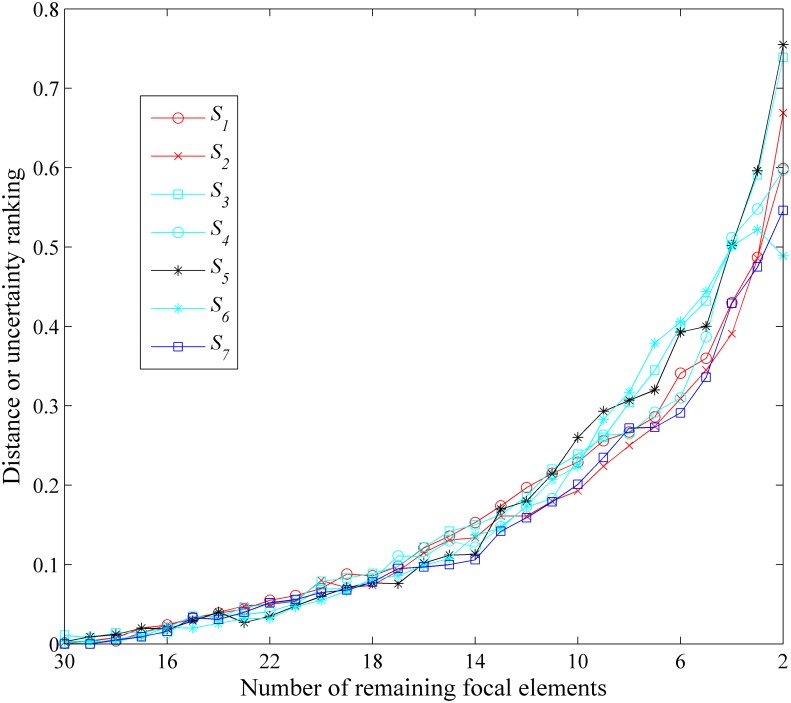
Comparisons in terms of order-preservation for uncertainty. Evaluation of distortion caused by the approximation in terms of uncertainty degree.

The above simulation procedure is repeated 500 times (in each run, 5 BBAs are re-generated randomly), the different approximation *S*_*i*_’s averaged distances of ordering at different *k* are listed in Fig 5.

The average distance of ordering over all *k* for different approximations are listed in Table 14.

**Table 14:** Averaged distance of ordering over all *k* values.

BBA approximations	Distance of ordering
*S*_1_	0.1679
*S*_2_	0.1576
*S*_3_	0.1874
*S*_4_	0.1667
*S*_5_	0.1808
*S*_6_	0.1698
*S*_7_	0.1513

As we can see in the Fig 5 and Table 14 that our newly proposed approach usually has the smallest average distance between two orderings, which represents the high capability in order-preservation, i.e., our new approach only causes the relatively small change to the order of the uncertainty degree. As aforementioned, this also represents the less loss of information and less distortion of relation caused by the approximation.

#### Simulation III—Comparisons in terms of probabilistic decision preservation

In this Simulation III, we compare all the BBA approximations in terms of the probabilistic decision preservation. It is hard to make the decision based on the original BBA and the decision based on the approximated one always have the same results, therefore, we calculate the percentage of the probabilistic preservation for *S*_*i*_ as follows.

1. Randomly generate 1000 BBAs. One can also select 1000 BBAs out of the 10000 generated BBAs in the data set (S1 File).
2. Make probabilistic decision for the 1000 different original BBAs.
3. Apply the BBA approximation *S*_*i*_ to the 1000 original BBAs.
4. Make probabilistic decision for the 1000 different approximated BBAs.
5. Count the number *N*^*i*^ of cases where the probabilistic decision results for the original BBA and its corresponding approximated BBA using *S*_*i*_ are the same.
6. Output the percentage *Pd*(*i*) = *N*^*i*^/1000 × 100%.

**Fig 6:**
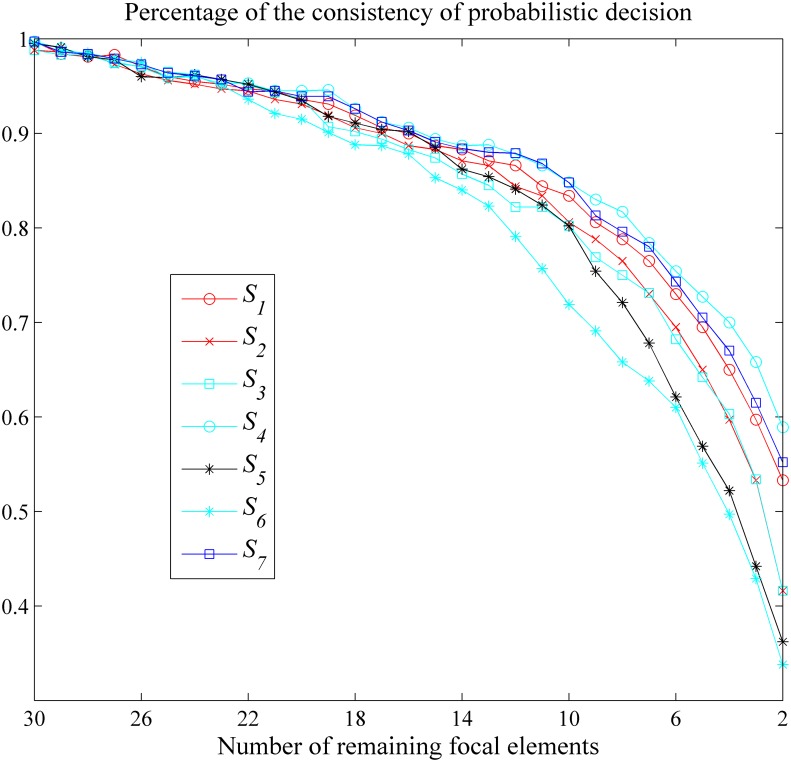
Comparisons in terms of probabilistic decision consistency. Evaluation of the distortion in terms of the probabilistic decision.

The random generation of BBAs is according to Algorithm 1 in Table 13. A BBA approximation *S*_*i*_ with higher *Pd*(*i*) is more preferred. In this simulation, the FOD Θ is with cardinality of 5. The simulation results (i.e., the percentage of probabilistic decision preservation for various BBA approximations with preset different remaining focal elements’ number *k*) are shown in Fig 6.

The average consistency rate over all *k* for different approximations are listed in Table 15.

**Table 15:** Averaged probabilistic decision consistency rate over all *k* values.

BBA approximations	Distance of ordering
*S*_1_	0.8626
*S*_2_	0.8432
*S*_3_	0.8396
*S*_4_	0.8773
*S*_5_	0.8271
*S*_6_	0.8039
*S*_7_	0.8732

As we can see in Fig 6 and Table 15 that the percentage of the probabilistic decision results preservation of the rank-level fusion based approximation and our newly proposed approach is usually highest (or the 2nd highest). It means that our new approach has higher possibility to keep the probabilistic decision results before and after the approximation unchanged given preset number of remaining focal elements, which represents the less loss of information from a different angle and the less of distortion of the relation caused by the approximation.

#### Simulation IV—Comparisons in terms of plausibility preservation

In this simulation IV, we compare all the BBA approximations in terms of the probabilistic decision preservation.

1. Randomly generate 1000 BBAs according to Algorithm 1.
2. For each BBA *m*_*j*_, *j* = 1, …, 1000, calculate its corresponding plausibilities and generate the ordering $\Lambda_{Pl}^{i}$.
3. After applying a BBA approximation *S*_*i*_, calculate the corresponding plausibilities and generate the ordering $\Lambda_{Pl^{\prime }}^{j}$.
4. Calculate the distance between $\Lambda_{Pl}^{j}$ and $\Lambda_{Pl^{\prime }}^{j}$ denoted by $d_{i}^{j}$, *j* = 1, …, 1000.
5. Output the average distance $d_{i}=\sum_{j=1}^{1000}d_{i}^{j}/1000$.

The *S*_*i*_ with smaller *d*_*i*_ value is more preferred.

**Fig 7:**
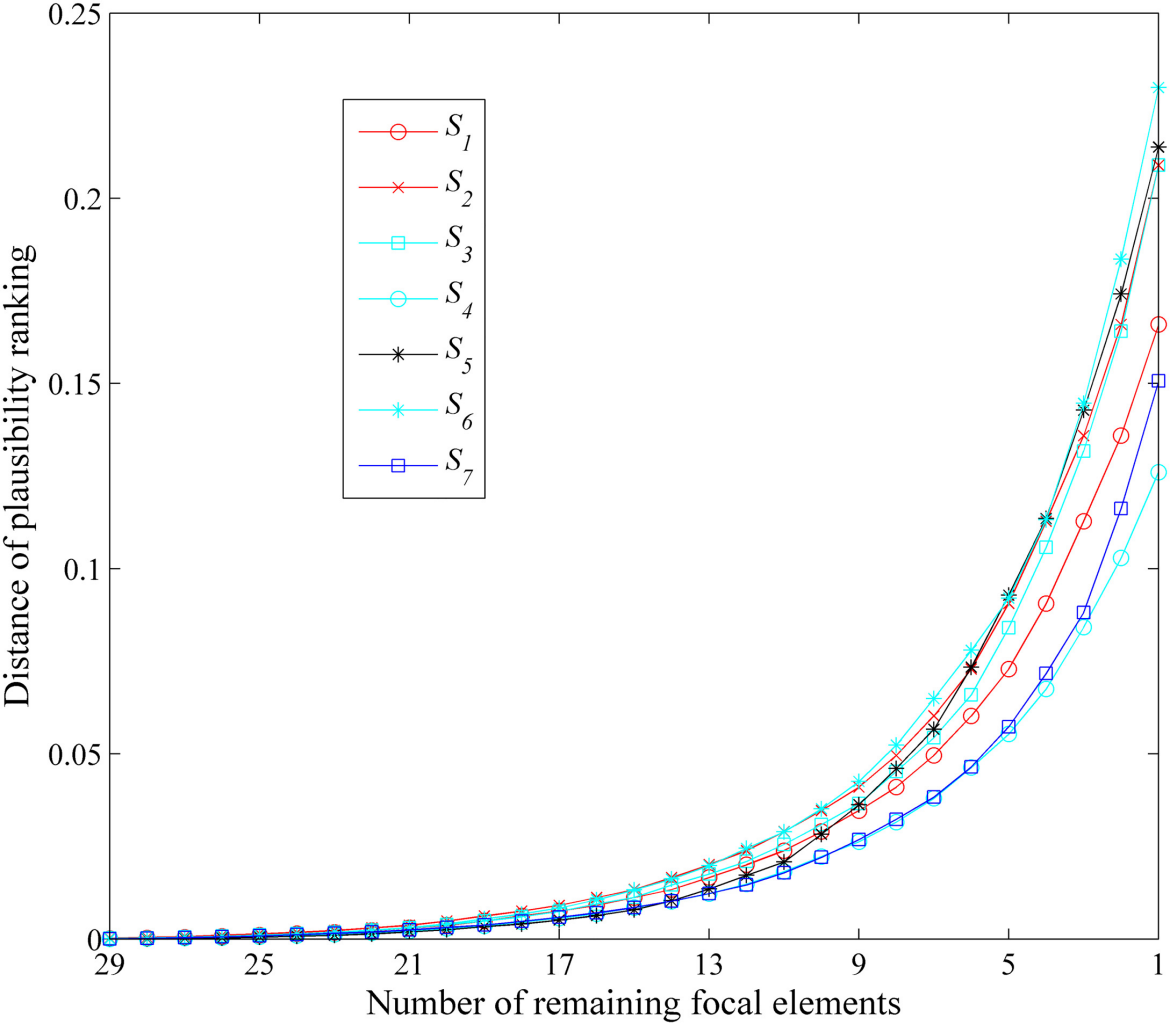
Comparisons in terms of order-preservation for plausibilities. Evaluation of the distortion caused by the approximation in terms of plausibilities.

The average distance of ordering over all *k* for different approximations are listed in Table 16. the different approximation *S*_*i*_’s averaged distances of ordering at different *k* are listed in Fig 7.

**Table 16:** Averaged distance of ordering over all *k* values.

BBA approximations	Distance of ordering
*S*_1_	0.0318
*S*_2_	0.0390
*S*_3_	0.0366
*S*_4_	0.0240
*S*_5_	0.0371
*S*_6_	0.0409
*S*_7_	0.0256

As we can see in the Fig 7 and Table 16 that the rank-level fusion based approximation and our newly proposed approach usually has the smallest average distance between two orderings, which represents the high capability in order-preservation for the plausibilities, i.e., our new approach only causes the relatively small change to the order of the plausibilities of events. It represents the less distortion caused by the BBA approximation.

## Conclusions

A novel iterative BBA approximation approach based on the distance of evidence and two new evaluation approaches for BBA approximations are proposed in this paper. The new approximation can effectively simplify the BBA, and is with less loss of information. It also makes a good balance between the precision and the tractability of the approximation. Two new performance evaluation approaches for BBA approximations are related to the uncertainty order-preservation and the consistency of probabilistic decision, respectively. Simulations and experiments are provided to illustrate and support our new BBA approximation and related evaluation approaches.

In this paper, we use the distance of evidence (closeness) to measure the loss of information in the BBA approximation. The closeness between two BBAs has strong correlation with the difference of information in two BBAs, thus, the closeness can be used to represent the loss of information. However, to be more strict, they are two different concepts. In our future work, we will try to directly use the difference between the degrees of uncertainty for BBAs (not the difference between the orders of uncertainty degree as we proposed in the uncertainty preservation) to represent the loss of information. The problem is how to design or select comprehensive uncertainty measures for BBAs, because the current total uncertainty measures in evidence theory including AU used in this paper and the ambiguity measure (AM) [27] are designed by generalizing the uncertainty measures in the probabilistic framework. They all have limitations [27, 28] and cannot always comprehensively describe the uncertainty in a BBA. Furthermore, although we propose two evaluation approaches for BBA approximations, how to evaluate the BBA approximation is still an opening and challenging problem. More solid, especially quantitative performance evaluation approaches are our research focus in future.

## Supporting Information

S1 File 10000 BBAs for test. Totally 10000 different BBAs for testing different approximation approach.
(MAT)
